# 3’UTR polymorphism of Thymidylate Synthase gene increased the risk of persistence of pre-neoplastic cervical lesions

**DOI:** 10.1186/s12885-020-06811-7

**Published:** 2020-04-15

**Authors:** Nayara Nascimento Toledo Silva, Ana Carolina Silva Santos, Verlândia Mendes Nogueira, Cláudia Martins Carneiro, Angélica Alves Lima

**Affiliations:** 1 Programa de Pós-Graduação em Ciências Farmacêuticas, Departamento de Análises Clínicas, Escola de Farmácia, Universidade Federal de Ouro Preto, Campus Morro do Cruzeiro, Ouro Preto, Minas Gerais 35400-000 Brazil; 2 Centro Estadual de Atenção Especializada (CEAE) de Itabirito, Rua Antônio Carlos, 202 - Praia, Itabirito, Minas Gerais 35450-000 Brazil; 3 Programa de Pós-Graduação em Ciências Biológicas e Biotecnologia, Departamento de Análises Clínicas, Escola de Farmácia, Universidade Federal de Ouro Preto, Campus Morro do Cruzeiro, Ouro Preto, Minas Gerais 35400-000 Brazil

**Keywords:** Genetic polymorphisms, Thymidylate synthase, TS3’UTR, Pre-neoplastic cervical lesions, HPV

## Abstract

**Background:**

Cervical cancer is caused by high-risk Human Papillomavirus (hr-HPV) infection associated with cofactors that has been analyzed as predictors of the remission or persistence of cytological abnormalities remission or persistence. These cofactors can be either environmental, epigenetic, or genetic. Polymorphism in genes of enzymes that act on one-carbon metabolism alter their activity and also may be associated with cervical carcinogenesis because they affect DNA synthesis and repair, and gene expression. Therefore, this study aimed to analyze the risk of persistence of pre-neoplastic cervical lesions according to genetic polymorphisms involved in one-carbon metabolism.

**Methods:**

Our sample consisted of 106 women, divided into two groups – Remission (*n* = 60), i.e., with the presence of pre-neoplastic lesions at first meeting (T_1_) and normal cytology after 6 months of follow-up (T_2_), and Persistence (*n* = 46), i.e., with the presence of pre-neoplastic lesions at T_1_ and T_2_. We obtained cervical samples for cytological analysis (T_1_ and T_2_), HPV detection (T_1_), and evaluation of polymorphism C667T of Methylenetetrahydrofolate Reductase (MTHFR C677T), A2756G of Methionine Synthase (MS A2756G), A66G of Methionine Synthase Reductase (MTRR A66G), double or triple 28 bp tandem repeat in 5′-untranslated enhanced region of Thymidylate Synthase (TSER), and 6 bp deletion at nucleotide 1494 in TS 3′-untranslated region (TS3’UTR). To analyze all genetic polymorphisms simultaneously, we calculated the Genetic Risk Score (GRS).

**Results:**

We observed no differences between the Remission and Persistence groups regarding the GRS. Also, there were no differences in the genotypic and allelic distribution of MTHFR C677T and MS A2756G polymorphisms. However, the risk of persistence was higher among women with the heterozygote genotype - ins/del [OR (IC95%): 3.22 (1.19–8.69), *p* = 0.021], or the polymorphic genotype – del/del [OR (IC95%): 6.50 (1.71–24.70), *p* = 0.006] of TS3’UTR. Conclusions: The presence of the TS3’UTR polymorphism increased the risk of persistence of cervical abnormalities. This genetic variant could be a potential marker of cervical carcinogenesis and therefore assist the follow-up of women with persistent pre-neoplastic cervical lesions.

## Background

Persistent high-risk Human Papillomavirus (hr-HPV) infection is the main cause of cervical cancer. However, only 10% of the women infected by hr-HPV develop pre-neoplastic lesions and less than 1% progress to cervical cancer. Only approximately 30% of high-grade cervical lesions progress to uterine cervix cancer, and spontaneous regression occurs in 20–40% of the cases [1–4]. Thus, the risk of cervical carcinogenesis depends on hr-HPV infection and host-dependent features [5, 6]. Environmental, genetic, and epigenetic cofactors of cervical carcinogenesis has been analyzed as markers for diagnosis, prognosis, and for auxiliary the treatment of pre-neoplastic cervical lesions since they could be predictors of cytological abnormalities remission or persistence [7–10].

Several genetic alterations characterize cervical cancer, such as genomic instability, chromosomal aberration, and the integration of the HPV DNA into the host genome [4, 7]. These alterations may impact the risk of cervical carcinogenesis substantially. Polymorphism in the genes of enzymes that act on one-carbon metabolism alter their activity and may be associated with cervical carcinogenesis [11–13]. Some of these enzymes are Methylenetetrahydrofolate Reductase (MTHFR), Methionine Synthase (MS), Methionine Synthase Reductase (MTRR), and Thymidylate Synthase (TS).

The MTHFR enzyme, whose gene is located on chromosome 1p36.3, is a flavoprotein that acts on folate metabolism and is essential for DNA integrity [14]. MTHFR C677T polymorphism consists of the exchange of cytosine (C) for thymine (T) at nucleotide 677. Such polymorphism results in the substitution of Alanine for Valine, which leads to a decrease in MTHFR activity [14, 15]. This Single Nucleotide Polymorphism (SNP) can change the susceptibility to carcinogenesis by modulating the availability of 5,10-methyleneTHF at different points in folate metabolism [16].

MS is a vitamin B12-dependent enzyme, essential to the maintenance of intracellular folate levels; it also catalyzes the methylation of homocysteine to methionine [17, 18]. What causes the MS A2756G polymorphism is the exchange of adenine (A) for guanine (G) at nucleotide 2756 – which results in the substitution of Aspartic Acid for Glycine, close to the binding domain of vitamin B12 [19, 20]. Van Der Put et al. (1997) suggested that this SNP affects the secondary structure of MS and has functional consequences. A study has demonstrated the association between the polymorphic allele (G) of MS and the reduction of the number of hypermethylated CpG islands in tumor suppressor genes [21]. Thus, MS A2756G may alter the activity of tumor suppressor genes, which would explain its association with the development of several types of tumors [22].

MTRR catalyzes the methylation of vitamin B12, which is a cofactor of MS enzyme [23]. A66G polymorphism of MTRR enzyme (MTRR A66G) leads to the exchange of A for G in the nucleotide 66. It also leads to the substitution of Isoleucine by Methionine, which results in a decrease of MTRR affinity by MS [24]. Thus, the polymorphic genotype was negatively associated with homocysteinemia, which alters DNA methylation and, consequently, gene expression [23, 25].

TS enzyme catalyzes the conversion of deoxyuridine monophosphate (dUMP) into deoxythymidine monophosphate (dTMP), the only de novo source of thymidine for DNA synthesis and repair. TS binds to RNA to repress the translation of its messenger RNA (mRNA) or other proteins and can regulate cell cycle progression [26–28]. Moreover, TS expression is an index of cell proliferation and the biological malignancy of cancer [29]. The most frequently studied polymorphisms are double or triple 28 bp tandem repeat in 5′-untranslated enhanced region (TSER), and 6 bp deletion/insertion at nucleotide 1494 in TS 3′-untranslated region (TS3’UTR). These two genetic variations may influence the TS gene expression and the stability of its mRNA, respectively [28].

Alterations in the one-carbon metabolism compromise the integrity of genetic material. Such compromise is due to changes in the nucleotide pool and uracil incorporation, which leads to DNA instability. Besides, global hypomethylation and site-specific hypermethylation are observed, which lead to the activation of proto-oncogenes and silencing of tumor suppressor genes [10, 30, 31].

Therefore, the aim of this study was to evaluat the risk of persistence of pre-neoplastic cervical lesions according to genetic polymorphisms involved in one-carbon metabolism.

## Methods

### Study design

Two hundred and eighty women were selected for this study between October 2016 and September 2018. They all lived in Minas Gerais State and attended the Basic Health Units of Ouro Preto and State Specialized Care Center of Itabirito. To be included in the study, their age should be at least 18 years. We excluded women who had been pregnant within the last 6 months, had a history of neoplasia, or the presence of cervical atypia in glandular cells.

**Fig. 1:**
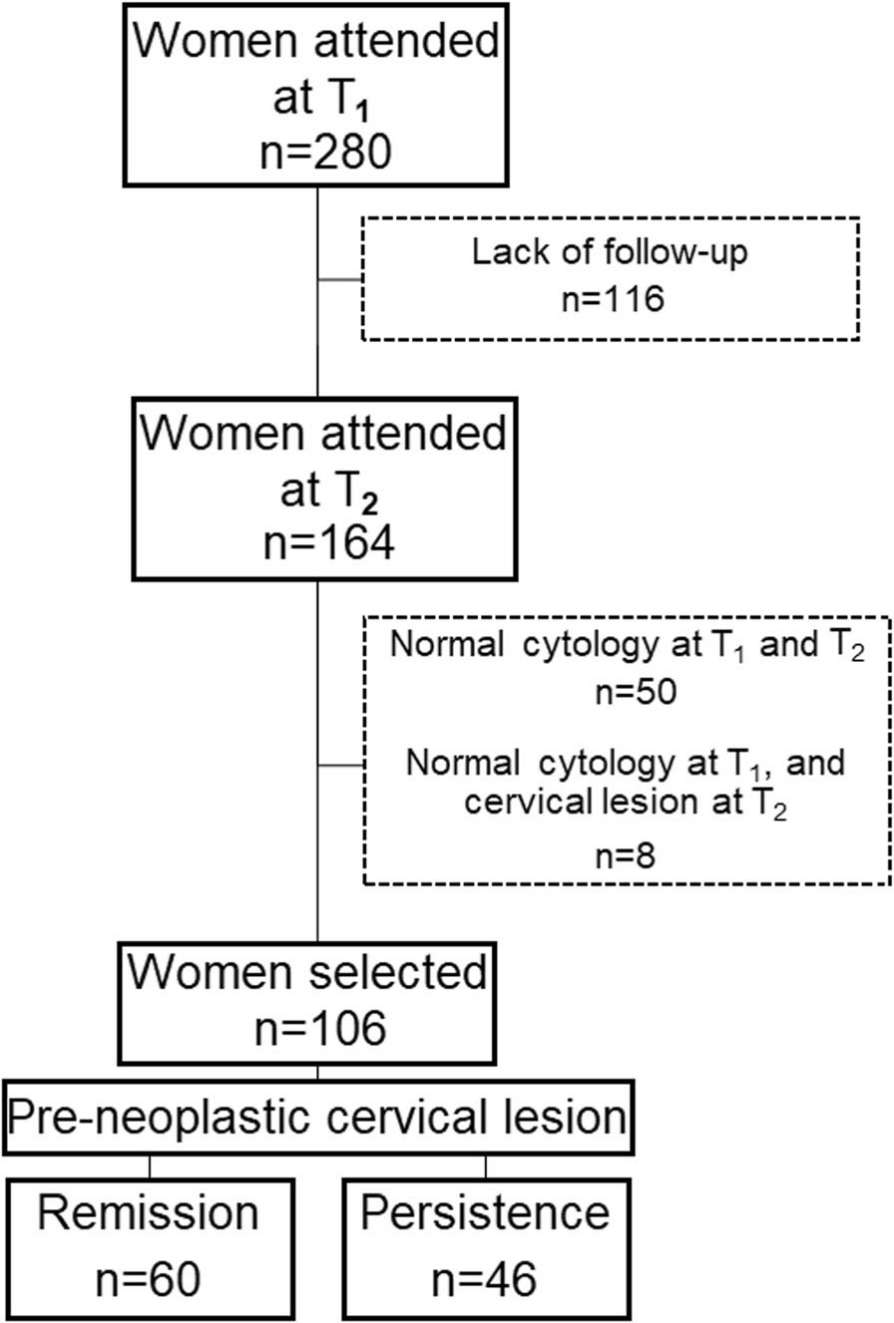
Study flow diagram

At the first meeting (T_1_), we interviewed them to get sociodemographic and behavioral information. We also collected a cervical sample for cytological analysis, genetic polymorphism evaluation, and HPV detection. After 6 months of follow-up (T_2_), 164 women performed the second cytological analysis. A total of 58 participants were excluded: 50 who presented normal cytology at T_1_ and T_2_, and eight who presented normal cytology at T_1_ and pre-neoplastic lesion at T_2_ (Fig. 1). We divided the resulting sample group (*n* = 106) into:

- Remission (*n* = 60): presence of pre-neoplastic lesion at T_1_, and normal cytology at T_2_, out of which;

- 45.0% (*n* = 27) presented Atypical Squamous Cells of Undetermined Significance (ASC-US) at T_1_;
- 35.0% (*n* = 21) presented Low-Grade Squamous Intraepithelial Lesion (LSIL) at T_1_;
- 15.0% (*n* = 9) presented High-Grade Squamous Intraepithelial Lesion (HSIL) at T_1_.
- 5.0% (*n* = 3) presented Atypical Squamous Cell – cannot exclude HSIL (ASC-H) at T_1_;

- Persistence (*n* = 46): presence of pre-neoplastic lesion at T_1_ and T_2_:

- 69.7% (*n* = 32) presented the same cervical lesion at T_1_ and T_2_:

- ASC-US at T_1_ and T_2_ (*n* = 8, 25.0%);
- LSIL at T_1_ and T_2_ (*n* = 9, 28.1%);
- ASC-H at T_1_ and T_2_ (*n* = 7, 21.9%);
- HSIL at T_1_ and T_2_ (*n* = 8, 25%).

b. 23.9% (*n* = 11) presented LSIL at T_1_ and ASC-US at T_2_;
c. 6.4% (*n* = 3) presented LSIL or ASC-US at T_1_ and ASC-H at T_2_.

T_1_: First meeting; T_2_: Second meeting; Remission: Presence of pre-neoplastic cervical lesion at T_1_, and normal cytology at T_2_; Persistence: Pre-neoplastic cervical lesion detected at T_1_ and T_2_.

We considered the presence of pre-neoplastic lesions when ASC-US, LSIL, HSIL, or ASC-H were detected. Also, as stated before, we only considered the Remission and Persistence groups for statistical analyses, not differentiating the types of cytological abnormalities.

The Research Ethics Committee of the Federal University of Ouro Preto approved this study (CAAE 57187316.7.0000.5150, and CAAE 88479718.0.0000.5150).

### Sample collection

Health care professionals obtained cervical samples through the conventional double collection, using Ayre spatulas for the ectocervical sample, and cylindrical brushes for the endocervical sample. After the confection of cervical smear for cytological analysis, the brush was conditioned in Phosphate-Buffered Saline (PBS) pH 7.2 and stored at − 80 °C for the evaluation of genetic polymorphisms.

### Cytological analysis

Cervical smears were stained according to the Papanicolaou method, and samples were evaluated based on cytomorphological criteria described in Bethesda System 2014 for reporting cervical cytological diagnoses (Nayar and Wilbur, 2015). Two cytopathologists evaluated all the samples. In the case of different results, a third professional evaluated the sample. The analyses were performed at the Laboratório de Análises Clínicas (LAPAC) from the Federal University of Ouro Preto.

### DNA extraction

DNA was extracted from the cervical samples with illustra blood genomicPrep Mini Spin™ Kit (GE Healthcare, Chicago, Illinois, USA). The evaluation of the quality and integrity of DNA was performed by amplification of the *β-actin* gene [32].

### HPV detection

HPV detection was performed by conventional Polymerase Chain Reaction (PCR) with MY09/MY11 primers, as described by Miranda et al. (2013). For positive samples, the HPV genotype was analyzed by Restriction Fragment Length Polymorphism (RFLP) [32]. HPV-negative samples were also analyzed by conventional PCR with GP5+/GP6+ primers [33].

### Genetic polymorphisms

MTHFR C677T (rs1801133), MS A2756G (rs1805087), MTRR A66G (rs1801394), and TS3’UTR (rs151264360) polymorphisms were evaluated by PCR-RFLP [14, 34–36]. TSER (rs34743033) was evaluated by PCR [37].

Sequences of primers, restriction enzymes, and PCR protocols are shown in *Supplementary Tables* *1**,**2**, and**3*. Table 1 shows the size of the DNA fragments that characterize the genotypes of the polymorphisms analyzed.

**Table 1:** Size of DNA fragments for genetic polymorphisms analysis

Genetic polymorphisms	Genotypes		
	No polymorphic	Heterozygote	Polymorphic
MTHFR C677T	198 bp	23 bp, 175 bp and 198 bp	175 bp and 198 bp
MS A2756G	211 bp	80 bp, 131 bp and 211 bp	80 bp and 131 bp
MTRR A66G	22 bp and 44 bp	22 bp, 44 bp and 66 bp	66 bp
TS3’UTR	70 bp and 88 bp	70 bp, 88 bp and 152 bp	152 bp
TSER	220 bp	220 bp and 248 bp	248 bp

GRS was calculated to evaluate the presence of all polymorphisms simultaneously, as described by Tomita et al. (2013). The presence of heterozygosity received one point; the presence of polymorphic homozygotes received two points. Non-polymorphic homozygotes were not scored (zero) [13]. Thus, the higher the GRS, the higher the frequency of genetic polymorphisms.

### Statistical analysis

Data were tabulated by Microsoft Office Excel™ (Microsoft, Redmond, Washington, USA), and analyzed by Statistical Package for the Social Sciences™ 17.0 (International Business Machines, New York, USA).

To evaluate the frequency of genotypes, we performed descriptive statistics. The allelic frequency was calculated by Genepop software [38]. HWE of genotypic frequencies was calculated by the HWE calculator, including analysis for ascertainment bias [39]. To compare the groups, we used the Chi-square. Also, we used binary logistic regression to calculate the relative risk (Odds Ratio) with a 95% confidence interval.

*p* values < 0.05 were considered evidence of statistically significant association.

## Results

To evaluate the association between genetic polymorphisms in enzymes involved in folate metabolism with cervical carcinogenesis, we analyzed samples of 106 women with remission or persistence of cytological abnormalities. We also evaluated sociodemographic and behavioral characteristics such as age, education, use of alcohol, smoking, marital status, information about sexual life, pregnancies, and use of hormonal contraceptives. These characteristics can be considered cofactors of cervical cancer [5, 8, 10, 40].

The mean age of participants was 39.7 ± 11.4 years, ranging from 19 to 71 years, and 32.1% (*n* = 34) were between 35 and 44 years. Most women resided in urban areas (*n* = 97, 91.5%), had family financial income <US$250/month (*n* = 70, 74.5%), high school education (*n* = 46, 47.9%), were non-smokers (*n* = 81, 84.4%), and ingested alcoholic beverages (*n* = 57, 59.4%).

Most of the women reported being married or having a fixed partner (*n* = 77, 80.2%), having had the first sexual intercourse at the age of 18 years or older (*n* = 53, 56.4%), and three or more sexual partners (*n* = 55, 57.3%). Also, most of them did not use hormonal contraceptives (*n* = 61, 63.5%) and had already been pregnant (*n* = 81, 84.4%) (Table 2). We observed these characteristics at a similar rate and with no significant association (*p* < 0.05) among the Remission and Persistence groups (Table 2).

**Table 2:** Sociodemographic and behavioral characteristics

Characteristics		Cytological abnormality		
	Total n (%)	Remission n (%)	Persistence n (%)	p
Age (years)				
< 25	12 (11.3)	7 (11.7)	5 (10.9)	0.302
25–34	21 (19.8)	9 (15.0)	12 (26.1)	
35–44	34 (32.1)	18 (30.0)	16 (34.8)	
45–54	28 (26.4)	17 (28.3)	11 (23.9)	
≥ 55	11 (10.4)	9 (15.0)	2 (4.3)	
Area				
Urban	97 (91.5)	54 (90.0)	43 (93.5)	0.524
Countryside	9 (8.5)	6 (10.0)	3 (6.5)	
Income per person (US$/month)^1^				
< 250	70 (74.5)	40 (72.7)	30 (76.9)	
250–500	21 (22.3)	12 (21.8)	9 (23.1)	0.333
≥ 500	3 (3.2)	3 (5.5)	0	
Education^2^				
Elementary school/Illiterate	44 (45.8)	23 (41.8)	21 (51.2)	0.341
High school	46 (47.9)	27 (49.1)	19 (46.3)	
University	6 (6.3)	5 (9.1)	1 (2.4)	
Smoker^2^				
No	81 (84.4)	48 (87.3)	33 (80.5)	0.365
Yes^a^	15 (15.6)	7 (12.7)	8 (19.5)	
Use of alcoholic beverage^2^				
No	39 (40.6)	22 (40.0)	17 (41.5)	0.885
Yes^a^	57 (59.4)	33 (60.0)	24 (58.5)	
Marital status^2^				
Married/Fixed Partner	77 (80.2)	40 (72.7)	37 (90.2)	0.073
Single	9 (9.4)	8 (14.5)	1 (2.4)	
Widow/Divorced	10 (10.4)	7 (12.7)	3 (7.3)	
Age at first vaginal intercourse (years)^1^				
< 18	41 (43.6)	25 (45.5)	16 (41.0)	0.670
≥ 18	53 (56.4)	30 (54.5)	23 (59.0)	
Lifetime sexual partners^2^				
1	23 (23.9)	13 (23.6)	10 (24.4)	0.936
2	18 (18.8)	11 (20.0)	7 (17.1)	
≥ 3	55 (57.3)	31 (56.4)	24 (58.5)	
Use of hormonal contraceptive^2^				
No	61 (63.5)	39 (70.9)	22 (53.7)	0.082
Yes	35 (36.5)	16 (29.1)	19 (46.3)	
Pregnancies^2^				
0	15 (15.6)	9 (16.4)	6 (14.6)	0.130
1	19 (19.8)	14 (25.5)	5 (12.2)	
2	26 (27.1)	10 (18.2)	16 (39.0)	
3	22 (22.9)	12 (21.8)	10 (24.4)	
≥ 4	14 (14.6)	10 (18.2)	4 (9.8)	
HPV infection	52 (49.1)			
Negative	54 (50.9)	37 (61.7)	15 (32.6)	0.003
Positive		23 (38.3)	31 (67.4)	

Concerning HPV, the main factor for cervical carcinogenesis [3], 50.9% (*n* = 54) of the participants were infected. The infection rate was higher in the Persistence group (*n* = 31, 67.4%) than in the Remission group (*n* = 23, 38.3%) (*p* = 0.003). We obtained similar results with the analysis of hr-HPV infection alone (*p* = 0.000) (data not shown).

Participants excluded due to absence of information: ^1^Twelve; ^2^Ten. ^a^Amount or frequency not determined. Remission: presence of pre-neoplastic lesion at T_1_, and normal cytology at T_2_; Persistence: pre-neoplastic lesion detected at T_1_ and T_2_.

Regarding the genetic polymorphisms evaluated, MTRR A66G genotypic frequencies were 10.4% (*n* = 11), 76.4% (*n* = 81), and 13.2% (*n* = 14) of AA, AG, and GG, respectively. The genotypic frequency of TSER was 35.8% (*n* = 38) of 2R/2R, 31.1% (*n* = 33) of 2R/3R, and 33.0% (*n* = 35) of 3R/3R. However, we could not find the distribution of genotypes of MTRR A66G and TSER of the Remission group under Hardy-Weinberg equilibrium (*p* = 0.000). Thus, these polymorphisms were excluded from further analyses in this study.

MTHFR C677T genotypic frequencies were 50.0% (*n* = 53) of CC, 45.3% (*n* = 48) of CT, and 4.7% (*n* = 5) of TT; the T allelic frequency was 27.4%. We detected the MS A2756G polymorphic genotype in 3.8% (*n* = 4) of the samples, and G allele in 19.8%. On the other hand, a higher frequency of women presented polymorphic genotype for TS3’UTR genetic variation (16.0%, *n* = 17), and the frequency of del allelic was 41.5% (Table 3).

**Table 3:** Frequencies of genetic polymorphisms according Remission or Persistence of pre-neoplastic cervical lesions

Genetic polymorphisms		Cytological abnormality			
	Total	Remission (*n* = 60)	Persistence (*n* = 46)	OR (IC95%)^a^	p
MTHFR C677T^1^					
*Genotype n (%)*					
CC	53 (50.0)	28 (46.7)	25 (54.3)	1.0	
CT	48 (45.3)	28 (46.7)	20 (43.5)	0.93 (0.40–2.12)	0.856
TT	5 (2.2)	4 (6.6)	1 (2.2)	0.25 (0.02–2.48)	0.245
*Allele %*					
C	72.6	70.0	76.1	1.0	
T	27.4	30.0	23.9	0.71 (0.34–2.11)	0.842
MS A2756G^2^					
*Genotype n (%)*					
AA	69 (65.4)	42 (70.0)	27 (58.7)	1.0	
AG	33 (31.1)	17 (28.3)	16 (34.8)	1.20 (0.50–2.89)	0.690
GG	4 (3.8)	1 (1.7)	3 (6.5)	4.99 (0.46–54.57)	0.188
*Allele %*					
A	80.2	83.3	76.1	1.0	
G	19.8	16.7	23.9	1.63 (0.59–4.53)	0.349
TS3’UTR^3^					
*Genotype n (%)*					
ins/ins	34 (32.1)	26 (43.3)	8 (17.4)	1.0	
ins/del	55 (51.9)	28 (46.7)	27 (58.7)	3.22 (1.19–8.69)	0.021
del/del	17 (16.0)	6 (10.0)	11 (23.9)	6.50 (1.71–24,70)	0.006
*Allele %*					
ins	58.5	66.7	47.8	1.0	
del	41.5	33.3	52.2	2.28 (1.00–5.22)	0.051
GRS n(%)					
≤ 2	77 (72.6)	48 (80.0)	29 (63.0)	1.00	
≥ 3	29 (27.4)	12 (20.0)	17 (37.0)	2.21 (0.89–5.48)	0.086

Hardy-Weinberg Equilibrium (HWE): ^1^p = 0.389; ^2^p = 0.625; ^3^p = 0.699. ^a^Adjusted for HPV infection. Remission: presence of pre-neoplastic lesion at T_1_, and normal cytology at T_2_; Persistence: pre-neoplastic lesion detected at T_1_ and T_2_.

To evaluate the association between the MTHFR C677T, MS A2756G, and TS3’UTR polymorphisms according to the course of cytological abnormalities, we compared the genotype distribution of the Remission and Persistence groups (Table 3).

There were no differences in the distribution of MTHFR C677T and MS A2756G and the course of cytological abnormalities (Table 3). On the other hand, women with persistent lesions had higher heterozygote and polymorphic genotypic frequencies of TS3’UTR than those from the Remission group. Furthermore, the ins/del and del/del genotypes increased the risk of persistence at least three times [OR (IC95%): 3.13 (1.21–8.12), *p* = 0.019; OR (IC95%): 5.96 (1.67–21.25), *p* = 0.006 - respectively] (Table 3).

To simultaneously evaluate the presence of MTHFR C677T, MS A2756G, and TS3’UTR, we determined the Genetic Risk Score (GRS) [13]. A score of ≥3 meant a high number of genetic variants. GRS ≥ 3 was more frequent in the Persistence group (*n* = 17, 37.0%) than in the Remission group (*n* = 12, 20.0%). Also, a high number of genetic variants presented higher risk of persistent lesions [OR (IC95%): 2.21 (0.89–5.48), *p* = 0.086] (Table 3). However, when adjusted for TS3’UTR, the risk of persistence according GRS was modified [OR (IC 95%): 1.26 (0.44–3.61), *p* = 0.669], showing that, between the three polymorphisms analyzed, only TS3’UTR was associated with the course of cytological abnormality.

## Discussion

Although persistent hr-HPV infection is the main cause of cervical cancer development, genetic alterations may impact on the risk of this neoplasia. Thus, genetic markers may be useful in the screening of pre-neoplastic and neoplastic cervical lesions, especially in cases of persistent HPV infections or recurrent cytological abnormalities [7, 41]. Moreover, the conventional methods used for screening cervical cancer cannot differentiate pre-neoplastic cervical lesions that will regress or persist and progress. Therefore, the prognosis of individual pre-neoplastic lesions should be predictable, to select women with a higher risk of persistence and progression, which could decrease the number of unnecessary treatment of lesions [4].

In this study, we evaluated five genetic polymorphisms in enzymes that act on one-carbon metabolism: MTHFR C677T, MS A2756G, MTRR A66G, TSER, and TS3’UTR. However, the distributions of MTRR A66G and TSER were not under Hardy-Weinberg Equilibrium (HWE), which led to their exclusion from further analyses. Other studies with the Brazilian population did not present a genotypic distribution of these polymorphisms under HWE either [42, 43].

Many polymorphisms were identified in the TS gene, located on chromosome 18p11.32. One of the most frequently studied polymorphisms is TS3’UTR, related to an increased in vitro degradation of its mRNA, which led to a decrease of expression protein [36]. The presence of the polymorphic allele (del) of TS3’UTR doubled the risk of persistence of cytological abnormalities. It was probably due to decreased synthesis of thymidylate, catalyzed by the TS enzyme. This enzyme’s activity is decreased by TS3’UTR polymorphism, and, as a result, DNA uracil is incorporated. The consequence of that is DNA instability and chromosome damage, crucial for carcinogenesis [26, 36, 44].

The integration of the HPV genome and the DNA of the host cell is probably one of the decisive steps for oncogenesis. It occurs preferentially in transcriptionally active regions, close to fragile DNA sites, and is observed in most invasive cancers [45]. Also, the HPV genome is integrated with chromosomes in some low-grade lesions and in most high-grade lesions [46, 47].

On the other hand, although the MTHFR C677T and MS A2756G polymorphisms have already been associated with pre-neoplastic and neoplastic lesions in the uterine cervix, we did not observe any association of these SNPs with the persistence of cytological abnormalities [11, 13].

The limitations of this study were the small sample size, the loss of participants during the study, and the short follow-up time. To better understand the role of genetic cofactors on cervical carcinogenesis, it is necessary to carry out more extensive studies, for a more extended period, and with different population groups. Besides, one should also consider different grades of cytological abnormalities separately.

In this study, we only considered the Remission and Persistence groups for statistical analyses due to the small number of samples of each subgroup. Still, analyzing these subgroups separately, there was no significant difference in genotypic or allelic frequencies of the evaluated polymorphisms (data not shown).

Nevertheless, this was the first study to evaluate the link between the risk of persistence of pre-neoplastic cervical lesions and the presence of genetic polymorphisms in enzymes that act on folate metabolism. Some authors have shown an association between the TS3’UTR polymorphism and esophageal, gastric, and breast cancers, although the results were controversial and varied with ethnicity [48–52]. However, studies on this polymorphism as a marker of cervical carcinogenesis have shown promising results, and further investigations are needed.

## Conclusions

The presence of the TS3’UTR polymorphism increased the risk of cervical abnormalities persistence. Thus, this genetic variant could be a potential marker of cervical carcinogenesis, which would assist the follow-up of women with persistent pre-neoplastic cervical lesions.

## Supplementary information

**Additional file 1: Table S1.** Sequences of primers, and restrictions enzymes used for analysis of genetic polymorphisms. **Table S2.** PCR protocol used for analysis of genetic polymorphisms. **Table S3.** Reagents for analysis of genetic polymorphisms by PCR.

## Data Availability

The datasets used and/or analyzed during the current study are available from the corresponding author on reasonable request.

## Abbreviations

A: Adenine
ASC-H: Atypical Squamous Cell – cannot exclude High Grade Squamous Intraepithelial Lesion
ASC-US: Atypical Squamous Cells of Undetermined Significance
C: Cytosine
dTMP: deoxythymidine monophosphate
dUMP: deoxyuridine monophosphate
G: Guanine
GRS: Genetic Risk Score
HPV: Human Papillomavirus
hr-HPV: high-risk Human Papillomavirus
HSIL: High Grade Squamous Intraepithelial Lesion
HWE: Hardy-Weinberg Equilibrium
LAPAC: Laboratório de Análises Clínicas
LSIL: Low Grade Squamous Intraepithelial Lesion
mRNA: messenger RNA
MS: Methionine Synthase
MS A2756G: polymorphism A2756G of Methionine Synthase
MTHFR: Methylenetetrahydrofolate Reductase
MTHFR C677T: polymorphism C667T of Methylenetetrahydrofolate Reductase
MTRR: Methionine Synthase Reductase
MTRR A66G: Polymorphism A66G of Methionine Synthase Reductase
PBS: Phosphate-Buffered Saline
PCR: Polymerase Chain Reaction
RFLP: Restriction Fragment Length Polymorphism
SNP: Single Nucleotide Polymorphism
T: Thymine
T_1_: First meeting
T_2_: Second meeting, after 6 months of follow-up
TS: Thymidylate Synthase
TS3’UTR: 6 bp deletion at nucleotide1494 in TS 3′-untranslated region
TSER: Double or triple 28 bp tandem repeat in 5′-untranslated enhanced region of Thymidylate Synthase
