# Supplementary material for: 3’UTR polymorphism of Thymidylate Synthase gene increased the risk of persistence of pre-neoplastic cervical lesions
Source: BMC Cancer. 2020 Apr 15;20:323. doi: 10.1186/s12885-020-06811-7 (PMC7161242; doi:10.1186/s12885-020-06811-7)
Supplement: Supplementary file 1 — Additional file 1: Table S1. Sequences of primers, and restrictions enzymes used for analysis of genetic polymorphisms. Table S2. PCR protocol used for analysis of genetic polymorphisms. Table S3. Reagents for analysis of genetic polymorphisms by PCR. [file 12885_2020_6811_MOESM1_ESM.docx]

**3’UTR Polymorphism of Thymidylate Synthase gene** **increased the risk of persistence of pre-neoplastic cervical lesions**

**Nayara Nascimento Toledo Silva^*^**

Programa de Pós-Graduação em Ciências Farmacêuticas, Departamento de Análises Clínicas, Escola de Farmácia, Universidade Federal de Ouro Preto. Campus Morro do Cruzeiro, Ouro Preto, Minas Gerais, Brazil. Zip Code: 35400-000.

E-mail: nayarants@gmail.com. *Corresponding author

**Ana Carolina Silva Santos**

Programa de Pós-Graduação em Ciências Farmacêuticas, Departamento de Análises Clínicas, Escola de Farmácia, Universidade Federal de Ouro Preto. Campus Morro do Cruzeiro, Ouro Preto, Minas Gerais, Brazil. Zip Code: 35400-000.

E-mail: aninhacarol_ss@yahoo.com.br

**Cláudia Martins Carneiro**

Programa de Pós-Graduação em Ciências Biológicas e Biotecnologia, Departamento de Análises Clínicas, Escola de Farmácia, Universidade Federal de Ouro Preto. Campus Morro do Cruzeiro, Ouro Preto, Minas Gerais, Brazil. Zip Code: 35400-000.

E-mail: carneirocm@gmail.com

**Verlândia Mendes Nogueira**

Centro Estadual de Atenção Especializada (CEAE). Rua Antônio Carlos, 202 - Praia, Itabirito, Minas Gerais, Brazil. Zip Code: 35450-000.

E-mail: verlandia.mendes@gmail.com

**Angélica Alves Lima**

Programa de Pós-Graduação em Ciências Farmacêuticas, Departamento de Análises Clínicas, Escola de Farmácia, Universidade Federal de Ouro Preto. Campus Morro do Cruzeiro, Ouro Preto, Minas Gerais, Brazil. Zip Code: 35400-000.

E-mail: aalimaufop@gmail.com

#### **TABLE 1**: Sequences of primers, and restrictions enzymes used for analysis of genetic polymorphisms.

| **Genetic polymorphisms** | **Sequences of nucleotides** | **Restriction Enzyme** | **Reference** |
| --- | --- | --- | --- |
| MTHFR C677T | 5’ TGAAGGAGAAGGTGTCTGCGGGA 3’  5’ AGGACGGTGCGGTGAGAGTG 3’ | *HinfI* | (14) |
| MS A2756G | 5’ TGTTCCAGACAGTTAGATGAAAATC3’  5’ GATCCAAAGCCTTTTACACTCCTC3’ | *HaeIII* | (48) |
| MTRR A66G | 5’GCAAAGGCCATCGCAGAAGACAT3’  5’GTGAAGATCTGCAGAAAATCCATGTA3’ | *NdeI* | (49) |
| TS3’UTR | 5’CAAATCTGAGGGAGCTGAGT3’  5’CAGATAAGTGGCAGTACAGA3’ | *DraI* | (36) |
| TSER | 5'GTGGCTCCTGCGTTTCCCCC3'  5'CCAAGCTTCGCTCCGAGCCGGCCACAGGCATGGCGCGG3' | NA^1^ | (50) |

^1^NA: Not applicable.

#### **TABLE 2:** PCR protocol used for analysis of genetic polymorphisms.

| **Genetic polymorphisms** | **Number of cycles** | **Temperature**  **(°C)** | **Time**  **(minutes)** | **Stage** |
| --- | --- | --- | --- | --- |
| MTFHR C677T | 1 | 94 | 1 | Denaturation |
|  | 35 | 94 | 1 | Denaturation |
|  |  | 59 | 0.5 | Annealing |
|  |  | 72 | 0.5 | Extension |
|  | 1 | 72 | 5 | Final Extension |
| MS A2756G | 1 | 95 | 2 | Denaturation |
|  |  | 95 | 1 | Denaturation |
|  | 35 | 48 | 1 | Annealing |
|  |  | 72 | 1 | Extension |
|  | 1 | 72 | 7 | Final Extension |
| MTRR A66G | 1 | 94 | 4 | Denaturation |
|  | 35 | 94 | 0.5 | Denaturation |
|  |  | 53 | 0.5 | Annealing |
|  |  | 72 | 0.5 | Extension |
|  | 1 | 72 | 5 | Final Extension |
| TSER | 1 | 94 | 4 | Denaturation |
|  | 35 | 94 | 1 | Denaturation |
|  |  | 60,6 | 1 | Annealing |
|  |  | 72 | 1 | Extension |
|  | 1 | 72 | 7 | Final Extension |
| TS3’UTR | 1 | 94 | 5 | Denaturation |
|  | 30 | 94 | 0.5 | Denaturation |
|  |  | 53 | 0.75 | Annealing |
|  |  | 72 | 0.75 | Extension |
|  | 1 | 72 | 5 | Final Extension |

#### **TABLE 3:** Reagents for analysis of genetic polymorphisms by PCR

|  | **Genetic polymorphisms** | | |  |  |
| --- | --- | --- | --- | --- | --- |
| **Reagents** | **MTHFR C677T** | **MS A2756G** | **MTRR A66G** | **TSER** | **TS3’UTR** |
| PCR Master Mix^™1^ | 12.5 μl | 12.5 μl | 12.5 μl | 12.5 μl | 12.5 μl |
| Primers^2^ (10 pmol/μl) | 0.25 μl | 1.0 μl | 1.0 μl | 1.0 μl | 1.0 μl |
| DNA (15 ng/μl) | 1.0 μl | 1.0 μl | 2.5 μl | 5.0 μl | 2.5 μl |
| Water^3^ | 11.0 μl | 9.5μl | 8.0 μl | 5.5 μl | 8.0 μl |

^1^0.2mM each deoxyribonucleotide (dNTP), 1.5 mM MgCl_2_ and 1.0 unit of Taq DNA (Promega, Madison, Wisconsin, USA); ^2^IDT (Coralville, Iowa, USA); ^3^Nuclease Free-Water (Promega, Madison, Wisconsin, USA).
